# Iterative Multiple Bounding-Box Refinements for Visual Tracking

**DOI:** 10.3390/jimaging8030061

**Published:** 2022-03-03

**Authors:** Giorgio Cruciata, Liliana Lo Presti, Marco La Cascia

**Affiliations:** Dipartimento di Ingegneria, University of Palermo, 90128 Palermo, Italy; giorgio.cruciata@unipa.it

**Keywords:** visual tracking, deep tracking, iterative bounding box refinement

## Abstract

Single-object visual tracking aims at locating a target in each video frame by predicting the bounding box of the object. Recent approaches have adopted iterative procedures to gradually refine the bounding box and locate the target in the image. In such approaches, the deep model takes as input the image patch corresponding to the currently estimated target bounding box, and provides as output the probability associated with each of the possible bounding box refinements, generally defined as a discrete set of linear transformations of the bounding box center and size. At each iteration, only one transformation is applied, and supervised training of the model may introduce an inherent ambiguity by giving importance priority to some transformations over the others. This paper proposes a novel formulation of the problem of selecting the bounding box refinement. It introduces the concept of non-conflicting transformations and allows applying multiple refinements to the target bounding box at each iteration without introducing ambiguities during learning of the model parameters. Empirical results demonstrate that the proposed approach improves the iterative single refinement in terms of accuracy and precision of the tracking results.

## 1. Introduction

Visual object tracking aims to automatically locate a target in subsequent frames, generally by estimating the bounding box that encloses the target on the image plane [1]. In contrast to the object detection problem, where instances of predefined object classes are located on an image, in object tracking the target is often located in a class-agnostic way by considering only the information provided in an initial frame (for instance, the frame where the target first appears). Although it has been widely studied, visual tracking remains a challenging problem in real-world scenarios due to target occlusions, pose and appearance changes, and illumination variations [2].

Nowadays, the best performance in visual tracking is achieved by employing deep learning [2,3]. MDNet, proposed by the authors of [4], is a tracking-by-detection and regression algorithm that classifies into target/background a set of bounding boxes sampled every frame around the last known target location. The bounding box with the highest classification confidence score is later adjusted through regression. The deep classification model is trained in a multi-domain way. Despite the fact that MDNet is not the most recent tracker, it still achieves state-of-the-art performance on the famous OTB benchmark [5]. MDNet has two main limitations: One is related to the sampling and classification at each frame of several bounding boxes to select the optimal one; the other limitation is related to the use of a regression model to refine the selected bounding box.

Recent works have tried to improve MDNet by formulating the search of the optimal bounding box either as an iterative process where a discrete sequence of bounding box refinements is predicted to locate the target, as done in [6,7,8,9,10], or as the problem of regressing the bounding box coordinates at each frame as in [11].

**Figure 1:**
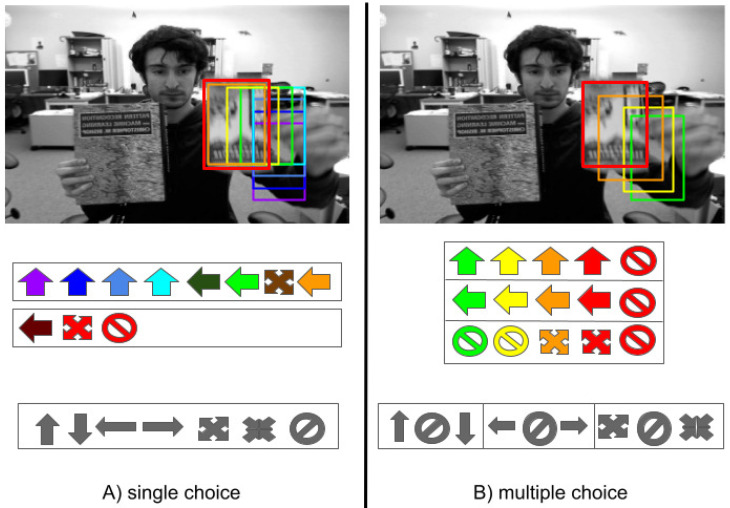
(**A**) In iterative single refinement, starting from an initial bounding box, a sequence of transformations to the bounding box is predicted and applied to locate the target. (**B**) With multiple refinements, a sequence of multiple non-conflicting transformations is predicted and applied.

In [6,12,13], at each frame, the bounding box is iteratively refined by applying shift (move right/left/up/down on the image plane) and scale (reduce/enlarge the bounding box size) refinements. The identity transformation is also included to account for the cases in which the bounding box must be accepted as it is. To implement such a strategy, the deep model takes as input the image patch corresponding to the currently estimated target bounding box, and provides as output the probability associated with each of the possible bounding box refinements. The one with the highest probability value is applied to the bounding box, and the process is iterated until a maximal number of iterations or the null transformation is selected. Figure 1A summarizes the process.

**Figure 2:**
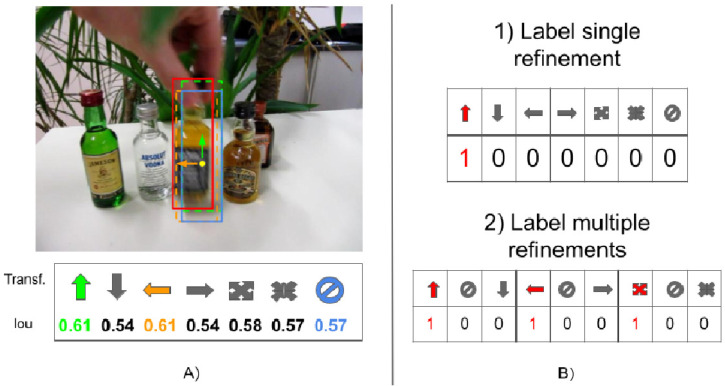
Image (**A**) shows the IoU values calculated after each of the possible refinements. Image (**B**) shows how 1-hot-encoding is used to annotate the data in single and multiple bounding box refinements.

This paper focuses on these kinds of approaches. We note that the above iterative process has several drawbacks. First, only one transformation can be applied at each iteration, thus leading to a higher computational cost to find the optimal bounding box. Second, the strategy introduces an ambiguity during the learning of the model parameters. Indeed, supervised training of the model is performed by providing the target patch and the type of bounding box refinement that should be applied to improve the tracking result. As shown in Figure 2, the effect of applying a transformation can be measured by estimating the intersection-over-union (IoU) value of the resulting bounding box and the ground-truth. Very often, more than a transformation would result in improved target localization (in Figure 2 the transformations “move up”, “move left”, and “enlarge”), but this is generally ignored by the supervised training procedure in [6,7,13]. Indeed, when using the 1-hot encoding schema to select the refinement, it has been given priority importance to a bounding box refinement over another by considering the first transformation with the highest IoU. One of the main contributions of this work is to allow the selection of multiple transformations. During training, multiple 1-hot encoding is used to indicate the transformations that might be applied to improve the bounding box.

Finally, we note that bounding box transformations may cancel each other. We call such refinements “conflicting transformations” (i.e., shifting the bounding box to the left and to the right). As reported in [13], refinements canceling each other may induce cycling behaviors.

In this paper, we deal with all the above issues by proposing a novel formulation of the problem of selecting the bounding box refinement (see Figure 1B). The contributions of this work can be summarized as follows:

- We introduce the novel concept of conflicting refinements and train the model to deal with them;
- We formulate the problem in such a way that multiple non-conflicting transformations can be applied at each iteration, leading to a speed up of the tracking process. As a consequence, a higher number of composite transformations can be handled by the model without any increases in its complexity;
- We limit the ambiguity during the training procedure by avoiding giving importance priority to some refinements over the others (see Figure 2).

To demonstrate our ideas empirically, we implemented our own tracking algorithm by adopting simple models and approaches common to several state-of-the-art tracking algorithms. We will make our tracker implementation publicly available to the scientific community. Our goal is not to achieve the highest accuracy and precision values in tracking. Instead, we aim to study the effect of formulating the problem of selecting the best target bounding box refinements in a different way.

In Section 2, we summarize the main characteristics of deep tracking approaches. In Section 3, we present our novel formulation of the problem. In Section 4, we provide details about the implementation we used to demonstrate our ideas. Finally, in Section 5, we discuss our contribution and contrast it with the state-of-the-art on the OTB benchmark. We also report the performance of our tracker implementation on the VOT benchmarks for completeness of results. Finally, in Section 6, we present conclusions and future work.

## 2. Related Work

Given a sequence of *T* images $\left\{F_{1};F_{2};\cdots F_{T}\right\}$, visual tracking is the problem of detecting the location $b_{t}$ on the image plane of a target moving in the environment over time or, more formally, estimating $b_{t}=\left(x_{t};y_{t}\right)$ with t∈[1;T] indexing the image frames. Often, $b_{t}$ also includes the width $w_{t}$ and height $h_{t}$ of the bounding box enclosing the target on the image. Many methods have been proposed in literature for tracking by using traditional approaches like linear dynamical systems, Kalman Filter [14,15], re-identification and data association [16,17], and correlation filter [18] together with hand-crafted features [2,19]. Recently, deep learning has proven to be particularly effective in extracting features for the recognition and detection of objects [20,21], and has been used to solve visual tracking problems [2]. In the following, we first briefly describe the different neural architectures and strategies commonly adopted in visual tracking. Then, we discuss methods that refine iteratively the target bounding box.

### 2.1. Network Architectures and Output

In general, it is possible to identify two categories of networks used in visual tracking: template-based and template-free neural networks. The former, such as the one proposed in [22], are composed of two or more branches that usually process the target template and the search area, and generally run in real-time without online parameter adaptation. These methods aim at locating the target depicted in the template image within the search area image. To account for target appearance changes, the template is in general updated online. The latter, such as the one proposed in [4], are composed of one branch. These methods do not need any templates but require online model adaptation to account for the target’s appearance changes. As online parameter adaptation is computationally costly, these models are generally slower but more accurate.

Regardless of the adopted neural architecture, three main strategies are used: tracking-by-detection, tracking-by-regression, and tracking-by-detection and regression. Tracking-by-detection approaches [23,24] are classification methods, which aim to discriminate between target and background. A large number of candidate target bounding boxes are drawn around the last known target location. The one yielding to the highest classification score is selected. As a result, performance is closely linked to the sampling strategy.

Tracking-by-regression approaches [22,25] use regression to locate the bounding box in subsequent frames by minimizing an objective function such as least-squared error.

Tracking-by-detection and regression methods [4,6,26,27,28] are hybrid approaches whose goal is detecting the most similar region to the target, and then refining the region through regression.

Recent approaches [29,30,31,32] might be included in the class of tracking-by-segmentation. These methods aim at tracking objects while producing a mask of the target object for each frame of the processed video. The work in [33], which focuses on the transformation of appearance features into motion-attentive representations, is also related.

Our work focuses on tracking-by-detection and regression. The method refines the bounding box by discrete transformations as in [6,7]. As one refinement of the bounding box cannot be enough to detect the target, refinements are made in an iterative way on the same frame. At each iteration, the tracking model not only predicts the refinement to apply, but also classifies the bounding box image into target/background and returns a confidence score as in tracking-by-detection strategies. This score is used to detect drifting of the tracker and decide whether to adopt a re-detection method purely based on tracking-by-detection strategies. In this sense, at each iteration, the model has to decide whether to regress the bounding box (by applying discrete transformations) or re-detect the target (by classification of multiple candidate bounding boxes).

### 2.2. Iterative Bounding Box Refinements

When iterative bounding box refinements are used, at each iteration the candidate target bounding box is transformed such that the target object is progressively more and more at the center of the image patch [12,13]. Numerous works in tracking used this formulation to find the bounding box of the tracked object [6,7,8,9,10]. The problem can be formulated as a Markov Decision Process and, while reinforcement learning is often used, these models generally require a supervised training procedure.

Often, the set of possible refinements are treated as categories and the model predicts the probabilities associated with each of them. When preparing the training set, given a target bounding box and its perturbed version, it is necessary to associate the transformation that the network has to predict when the perturbed bounding box is given in input. Among all transformations, the one maximizing the IoU of the transformed bounding box and the ground-truth is chosen.

In  [13], the method for object detection in [12] is adapted to perform visual tracking. Given an initial detection of the target, a pre-trained deep network (derived from the VGG-16 model in [34]) is used to extract features from the bounding box. The deep model is trained to shift and scale the bounding box to recenter the target. It has been noted that the model may sometimes start to select pairs of bounding box transformations that cancel each other. To overcome this issue, the bounding box is randomly perturbed whenever cycling behavior is detected.

In the work proposed in [6], an action-decision network (ADNet) is used to predict a probability distribution over the possible bounding box transformations. The network takes as input the cropped target image and the history of the last 10 selected bounding box transformations represented by a one-hot encoding schema. The network is pre-trained in a supervised way and also estimates a confidence score to decide when the tracking process must be re-initialized. At test time, a supervised adaptation of the latest fully connected layers is performed to make the model more robust to appearance changes. ADNet was later improved in IADNet [35]. During training, a multi-domain learning strategy [4] is used. An online adaptive update strategy based on meta-learning is used to estimate the most appropriate model parameters such that the parameters are closer to the optimal ones.

The work proposed in [7], RDNet, adopts two Siamese network-based models receiving as input the crop of the image corresponding to the currently estimated bounding box and a search region image both obtained from the current frame. The first model chooses among different shifting transformations; the second one selects a scaling transformation. Similarly to RDNet, we also treat shifting and scaling transformations separately, but we adopt one multi-branch network and formulate the problem as one selecting multiple non-conflicting transformations.

The model TSAS proposed in [36] uses a cascade of two networks to predict how to shift the target bounding box. The first network, trained in a supervised way, predicts the best bounding box transformation to locate the target; the second network assesses the quality of the predicted transformation. Finally, a regressor is used to refine the resulting bounding box. In our model, one branch provides the confidence of the model on the current tracking result and no postprocessing of the bounding-box is used to improve the results.

## 3. Multi-Refinements of the Bounding Box

At time *t*, the candidate target bounding box is defined as a 4-dimensional vector $b_{t}=\left[x_{t},y_{t},w_{t},h_{t}\right]$, where $\left(x_{t},y_{t}\right)$ is the center coordinates and $w_{t},h_{t}$ represent the width and height of the bounding box, respectively. Given an image frame $F_{t}$, the image patch $p_{t}$ is obtained by cropping $F_{t}$ based on the bounding box $b_{t}$ through the selection patch function $f_{b}\left(\cdot \right)$. The function can also include pre-processing steps to adapt the resulting image patch to the network input:

(1) $$p_{t}=f_{b}\left(b_{t},F_{t}\right).$$

Let *V* be the number of basic linear transformations that can be used to refine the bounding box *b*. These transformations define the space Φ of allowed discrete bounding box transformations. Let φ be a subset of *k* transformations $\varphi =\{\phi_{1},\phi_{2},\cdots ,\phi_{k}\}\subseteq \Phi$. A transformation θ(·)∈Φ is *conflicting* with the transformations in φ if

(2) $$b=\gamma_{i}\left(\theta \left(b\right)\right)$$

where $\gamma_{i}$ indicates any sequence of transformations in φ. In other words, bounding box changes operated through the refinement θ are canceled out by some of the refinements in φ.

If instead, for any sequences $\gamma_{i}$

(3) $$b\neq \gamma_{i}\left(\theta \left(b\right)\right),$$

then θ(.) is *non-conflicting* with the transformations in φ.

Keeping in mind the above definitions, we propose to split the bounding box refinements into *N* groups such that each transformation in one group is not in conflict with the transformations in all the other ones. We include within each of these groups a “null transformation”, namely, the identity transformation. In the following, the obtained groups are named *non-conflicting* groups.

As an example, let us consider the following discrete bounding box transformations: Φ= {*Left(Δ)*, *Right(Δ)*, *Up(Δ)*, *Down(Δ)*}, where the name of the transformation indicates the shifting direction of the bounding box, while Δ represents the number of pixels. A possible partition into two non-conflicting groups, $G_{h}$ and $G_{v}$, is $G_{h}=\left\{Left\left(\Delta \right),Right\left(\Delta \right)\right\}$ and $G_{v}=\left\{Up\left(\Delta \right),Down\left(\Delta \right)\right\}$.

### Output Layer for Non-Conflicting Refinements

**Figure 3:**
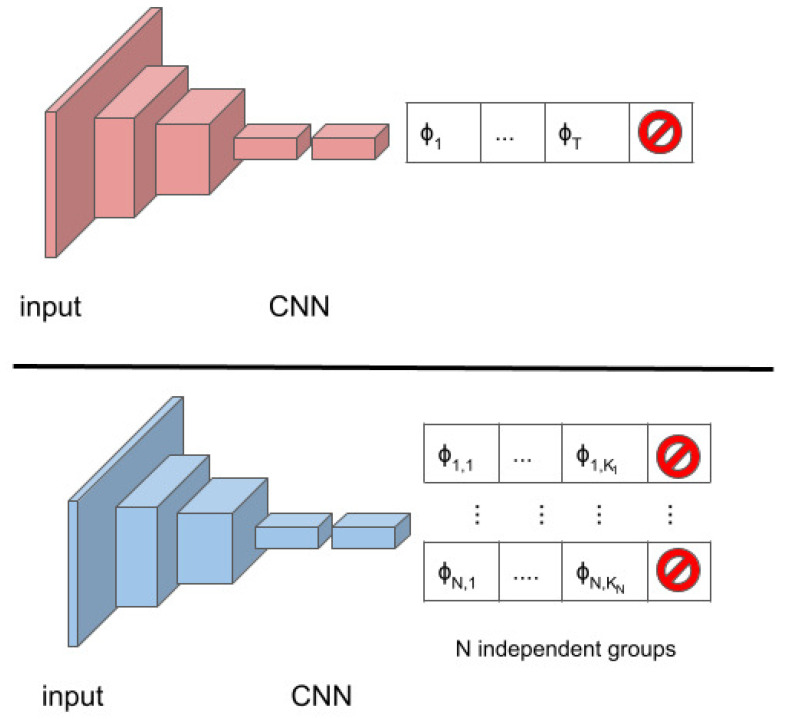
On the top, the model estimates the probability distribution over all the transformations. On the bottom, the model estimates *N* probability distributions, one for each group of transformations.

To deal with multiple non-conflicting refinements of the bounding box, we need to properly design the output layer of the deep model used within the tracking strategy. Figure 3 shows, on the top, the typical deep model used for iterative bounding box refinements [6,12,13]. At each iteration, the model takes as input an image patch and provides the probabilities of each of the *V* transformations $\phi_{i}\in \Phi$. Only the transformation with the highest probability is applied.

On the bottom, Figure 3 shows how the model needs be adapted to deal with *N* non-conflicting transformation groups. The network will provide *N* probability distributions over the $k_{i}$ transformations belonging to each group, with *i* varying in [1,N]. The distributions are computed independently from each other, and *N* different non-conflicting transformations are applied, one from each group.

## 4. Tracking by Iterative Multi-Refinements

**Figure 4:**
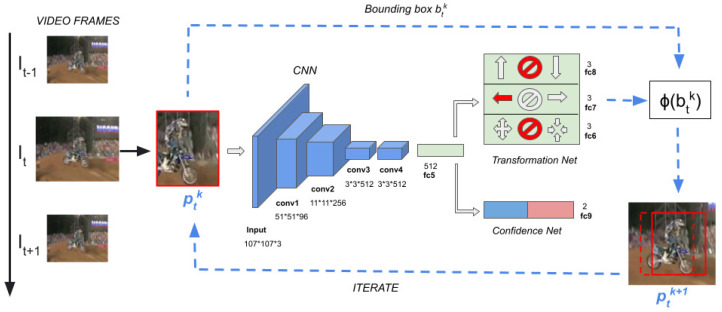
Architecture of our model. On the left, the arrow indicates the flow of frames. At time *t*, starting from the bounding box estimated at time *t*− 1, the method iterates a sequence of multiple transformations highlighted in red on the right side. The transformations are applied to the bounding box, which is used to get a new image patch to feed the model.

To demonstrate our idea, we implemented our own tracker. Our tracking architecture is template-free, and requires online fine-tuning of the parameters to adapt the model to the target appearance changes over time. As shown in Figure 4, our tracker takes advantage of one network with multiple output branches sharing the convolutional layers and the first dense layer. The first *N* output branches, namely, the subnet Transformation-Net, estimate *N* probability distributions over the refinements within the *N* non-conflicting groups of transformations. In the figure, N=3. The last output branch network, namely, the subnet Confidence-Net, provides a confidence score of the classification of the image patch into background/target.

At time *t*, starting from the target bounding box estimated at time t−1, our method uses the networks to estimate a sequence of linear transformations of the bounding box to locate the target in the image frame. The process ends when, for each non-conflicting transformation group, the null transformation is selected or a maximum number of iterations is reached.

The final confidence score is used to assess if the tracking failed, and in this case, a re-detection procedure based on particle filter is used. The entire tracking procedure is presented in Algorithm 1. In the following, we present details about the various steps performed by our tracker.
**Algorithm 1** Online tracking algorithm.**Input:** Pre-trained CNN ($w_{1}$,…,$w_{5}$)
            Initial target $b_{0}^{*}$
            Sequence of *V* frames $\left\{F_{0};F_{1};\ldots ;F_{t};\ldots ,F_{V}\right\}$
**Output:** Estimated sequence of target bounding-boxes ${\left\{b_{t}^{*}\right\}}_{t=1}^{V}$
1:Randomly initialize parameters $w_{6}$-$w_{9}$2:Draw training samples $X_{0}$ around $b_{0}^{*}$, $M_{S}.$push$\left(X_{0}\right)$, $M_{L}.$push$\left(X_{0}\right)$3:Update parameters $w_{4}$-$w_{9}$4:**for**t=1,⋯,V**do**5:    $b_{t}^{0}\leftarrow b_{t-1}^{*}$6:    **for** k=1,…,max_iter **do**7:        $p_{t}^{k}=f_{b}\left(b_{t}^{k-1},F_{t}\right)$8:        Select N refinements $\varphi_{i}$ based on Transformation-Net$\left(p_{t}^{k}\right)$9:        Apply $\varphi_{i}$ with i∈[1,N] to the bounding-box to estimate $b_{t}^{k}$10:    **end for**11:    Evaluate confidence score $q^{*}=$ Confidence-Net$\left(f_{b}\left(b_{t}^{k},F_{t}\right))\right)$12:    **if** $q^{*}>0.5$ **then**13:        $b_{t}^{*}\leftarrow b_{t}^{k}$14:        Draw sample $X_{t}$ around $b_{t}^{*}$15:        Update $M_{S}$ and $M_{L}$ by adding $X_{t}$ and limiting their size16:    **end if**17:    **if** $q^{*}<=0.5$ **then** (failure!) apply Re-detection to find $b_{t}^{*}$18:        Evaluate confidence score $q^{*}=$ Confidence-Net$\left(f_{b}\left(b_{t}^{*},F_{t}\right)\right)$19:    **end if**20:    **if** $q^{*}<=0.5$ **then** Update $w_{4}-w_{9}$ by using $M_{S}$21:    **else if** *t* mod 10=0**then** Update $w_{4}-w_{9}$ by using $M_{L}$22:    **end if**23:**end for**


### 4.1. Designing Non-Conflicting Refinements

In our implementation, each basic transformation depends on parameters calculated from the size of the current bounding box, similarly to what has been done in [6]. In particular, at the *k*-th iteration, we consider the values $\Delta w_{t}^{k}=\sigma \cdot w_{t}^{k-1}$ and $\Delta h_{t}^{k}=\sigma \cdot h_{t}^{k-1}$, where σ is a constant value equals to 0.03. These parameters represent, in pixels, the adjustments to the center coordinates or the width/height of the bounding box. Based on our experiment, considering the structure of the adopted CNN (a VGG-M), σ equals to 0.03 is the minimum value for the network to notice the effect of the transformations to the bounding box.

We also consider N=3 non-conflicting groups of refinements, each including two basic transformations. The first group, $G_{h}=\left\{Left\left(\Delta w_{t}^{k}\right),Right\left(\Delta w_{t}^{k}\right)\right\}$, shifts the bounding box to the left or to the right by adding/subtracting the value $\Delta w_{t}^{k}$ to the coordinate $x_{t}^{k-1}$.

The second group, $G_{v}=\left\{Up\left(\Delta h_{t}^{k}\right),Down\left(\Delta h_{t}^{k}\right)\right\}$, shifts the bounding box up or down by adding/subtracting the value $\Delta h_{t}^{k}$ to the coordinate $y_{t}^{k-1}$.

Finally, $G_{s}=\left\{Enlarge\left(\Delta w_{t}^{k},\Delta h_{t}^{k}\right),Reduce\left(\Delta w_{t}^{k},\Delta h_{t}^{k}\right)\right\}$, re-scales the bounding box to decrease or increase its size by adding/subtracting the value $\Delta w_{t}^{k}$ and $\Delta h_{t}^{k}$ to the bounding box width and height, respectively. Each of the above groups is augmented to also include the null transformation. These groups were created starting from the transformations used in ADNet [6], separating them into non-conflicting groups and excluding double shifts. The introduction of double shifts, namely, shifting transformations with a doubled value of σ, did not produce significant improvements in our experiments.

### 4.2. Deep Model

The structure of our network is described in Figure 4. Similarly to former approaches [4,6], the first four convolutional layers (conv1–conv4), with weights $\left(w_{1}-w_{4}\right)$, are taken from the VGG-M network [37]. The input layer is adapted to our input dimension and subsequent features maps; we used the same input size as in [4]. The fully-connected layer fc5, with weights $w_{5}$, is shared by both the Confidence- and Transformation-Net. Layers fc6–fc8, with weights $\left(w_{6}-w_{8}\right)$, provide the probability distributions for each of the transformation groups we defined and apply softmax activation functions. Layer fc9, with weights $w_{9}$, belongs to the Confidence-Net; with 2 units, it applies a softmax activation function.

### 4.3. Supervised Training of the Model

Offline training: An offline training procedure is used to learn the parameters $\left(w_{1}-w_{5}\right)$. This procedure is based on multi-domain learning [4] where layers (conv1-fc5) dealing with domain-independent information (such as motion blur, illuminations changes, and scale variations) and domain-specific layers (fc6–fc9) are treated differently. In particular, while the former are shared among all videos, the latter are initialized and trained for each video. The number of domains is equal to the number of videos contained in the training dataset. At each training iteration we use $X=[X_{c},X_{b}]$, where $X_{c}$ indicates the data used to train the Confidence-Net, and $X_{b}$ indicates the data used to train the Transformation-Net. The entire model is trained by alternating the training of the Transformation-Net (while freezing the Confidence-Net) and the training of the Confidence-Net (while freezing the Transformation-Net).

Online training: At test time, parameters $\left(w_{6}-w_{9}\right)$ are randomly initialized for each video sequence to be adapted online to the target appearance changes, and parameters $\left(w_{1}-w_{3}\right)$ are fixed and not trained online to speed up the online training, and to limit overfitting of the network. Online parameter adaptation is done every *s* frames (s=10) and, whenever a tracking failure has been detected, a re-detection step is performed. A failure is detected whenever the model predicts a confidence score lower than 0.5. Inspired by the works in [4,6], we update the parameters by using a long-term memory $M_{L}$ every *s* frames. This memory stores random samples from the last $mem_{L}=1000$ frames. In case of tracking failures, to speed up the model adaptation to the current target appearance, we update the parameters by using a short-term memory $M_{S}$. This memory stores random samples from the last $mem_{S}=20$ frames.

### 4.4. Sample Generation

Considering *N* groups of *k* transformations (including the identity one), there are overall $k^{N}$ composite transformations. In our implementation, $k^{N}=3^{3}=27$. To train the Transformation-Net, we used balanced mini-batches of 81 samples where the 27 composite transformations were equally represented. A grid sampling approach over the 4-dimensional space with a discrete uniformly distributed random step has been used.

To generate balanced mini-batches of 64 samples for training the Confidence-Net, we referred mainly to the technique used by [4]. We used the sampling methods reported in the public code, which slightly differs from the one described in the paper. The sampling is based on normal distributions whose mean and variance depend on the bounding box estimated at the previous iteration. If the sample comes from the first frame, it is considered positive if it yields to IoU>0.7. Otherwise, it is considered positive if the predicted confidence value is >0.5. Furthermore, we used hard negative sampling, meaning that we randomly selected a large number of negative samples from the short memory $M_{S}$ and select 32 samples with the highest positive classification score. This procedure improves the discriminative abilities of the Confidence-Net.

### 4.5. Implementation Details

Whenever a failure occurs, similarly to the works in [4,6], we sample 256 candidate target bounding boxes with the same schema adopted to generate mini-batches for training the Confidence-Net. The confidence score of these bounding boxes is evaluated, and the candidate with the highest score is selected as the predicted target bounding box.

As loss function, we adopted the categorical cross-entropy. The learning rate is fixed to 0.001 and the network is trained with SGD with a momentum of 0.9 and weight decay parameter of 0.0005. During offline training of the model, we used 287 domains, that is, 287 videos from the ALOV300 dataset [38]. In particular, for a number of 3 iterations, we sampled 5 frames from each video. For each frame we trained the model for 5 iterations. Training has been done alternating the domains.

At the first frame, layers (conv4-fc9) are trained for 30 iterations on samples generated based on the known target. The Transformation-Net is initialized by sampling 6 times all the possible composite transformations, for a total of 162 samples for each iteration. Online adaptation of the parameters was done for 10 iterations. During tracking, at each frame, 15 negative samples and all transformed bounding boxes with a confidence score >0.5 were stored in the long and short memories.

## 5. Experimental Results

Our goal, in this paper, is to demonstrate that iterative approaches to refine the bounding box have several drawbacks that can be overcome by allowing multiple non-conflicting refinements to the bounding box at each iteration. Therefore, we run two kinds of experiments: one to show the usefulness of our proposed approach, the other to compare our tracking strategy to state-of-the-art approaches on publicly available benchmarks. All experiments were conducted on a machine equipped with: 32 GB RAM, GPU RTX 2070 8GB RAM. Our prototype has been implemented in Python by using Tensorflow and runs at 5 fps on the GPU.

### 5.1. Single vs. Multiple Transformation Groups

We performed experiments by keeping the tracking strategy fixed and by varying the output layer of the deep model (single vs multiple transformation groups). As training of the model is important, we also test our approach by varying the model. To this purpose, we used the pre-trained parameters of the ADNet model [6], modified the last layer and compared single vs. multiple transformation groups. Finally, we tested how different training strategies of our model can affect the results. Experiments have been run on the OTB 100 benchmark [5].

**Figure 5:**
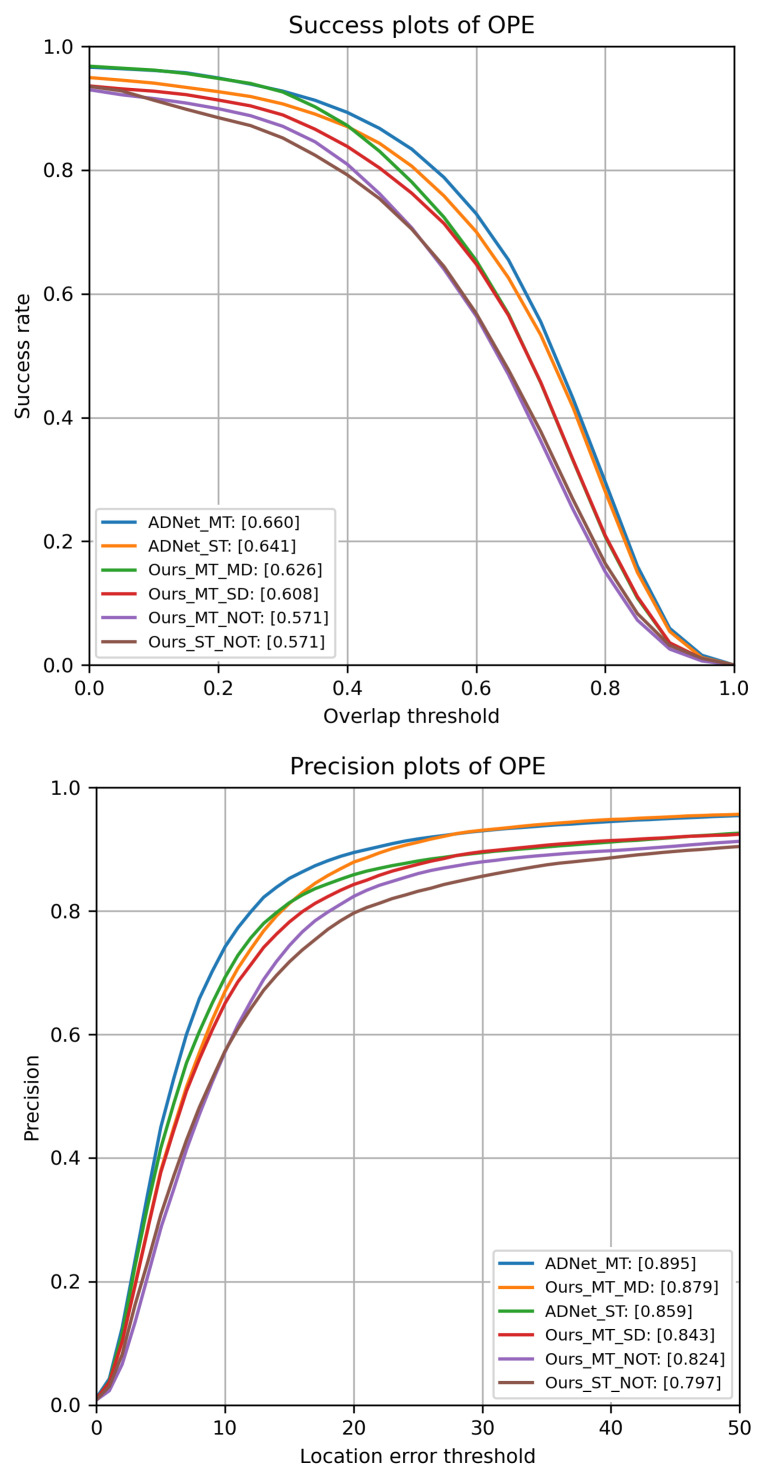
Success and Precision plots on OTB-100 (One-Pass Evaluation (OPE)). Overlap threshold and location error threshold indicate the threshold values used to compute the ROC curves. For the precision plot, the scores in the legend indicate the mean precisions when the location error threshold is 20 pixels. For the success plot, the scores indicate the area under curve (AUC).

All results are reported in Figure 5. Configurations with “NOT” directly use the initial parameters of VGG-M for the layers (conv1–4), while the fully connected layers (fc5–fc9) are initialized by random noises. Configurations with “ADNet” load the ADNet parameters.

For the above configurations, no offline training is performed and only online learning at test time is done.

Configurations with “MT” use multiple bounding box refinements, in contrast to “ST” where a single transformation is applied at each iteration. Finally, “MD” indicates that multi-domain learning is adopted to pre-train the model (offline learning), “SD” indicates a more classical training procedure where all videos are used to train all model layers.

As shown in Figure 5, models adopting the multiple bounding box refinements achieve higher performance than the corresponding ones with single refinements. However, the training strategy largely affects the performance of the method. Offline multi-domain learning allows achieving higher results. The method that yields to the highest performance uses ADNet parameters and the proposed refinement approach. We note that in terms of precision and success, we achieve higher results than the one published in [6] (0.88 and 0.646, respectively). On the other side, results with single refinements are almost identical, meaning that different implementation choices in the online tracking strategy may have little impact on performance.

Our offline-trained model differs from the ADNet especially because ADNet uses a reinforcement learning approach after the supervised training of the model.

### 5.2. Comparison on OTB and VOT

Table 1 shows the results achieved by our tracker in terms of precision (P), success (AUC), and frame rate (FPS). We used Got10K-toolkit [39] to run all the experiments. Compared to approaches using iterative bounding box refinements (in column Iter), such as TSAS [36] and ADNet, our model (Ours_MT_MD) achieves better/comparable results. Our ADNet-based tracker (ADNet_MT) achieves better/comparable results than those in [7,35,40], which also use iterative refinements.

**Figure 6:**
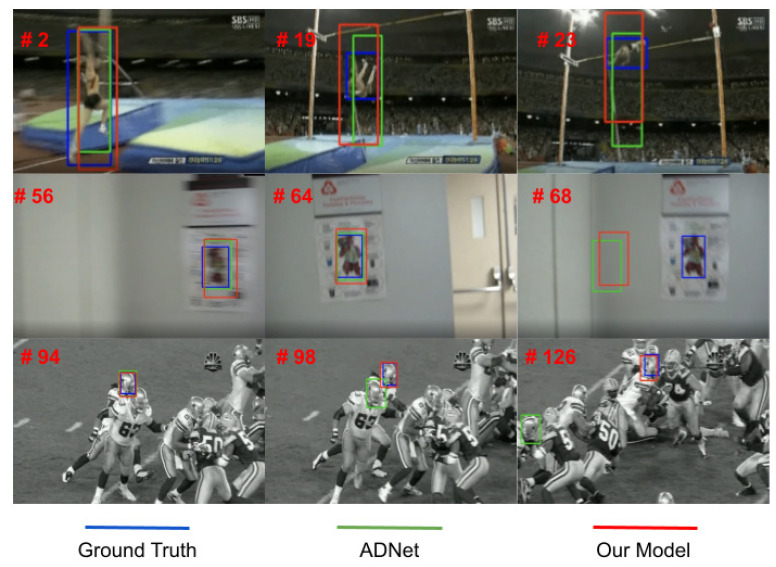
The figure shows some qualitative results of our tracker (red bounding-boxes) vs. ADNet (green bounding-boxes). Ground-truth is shown in blue.

Figure 6 shows some samples from three videos belonging to OTB. In the images at the first row, both our method and ADNet are unable to adapt to the actual target shape. This is because rescaling of height and width is done jointly and not separately. However, our method (red bounding-boxes) seems to center better the target. In the second row, both ADNet and our method are sensitive to large and abrupt camera motion. In the third row, where the target is among several instances of the same class (several players), ADNet drifts while our tracker is able to follow the target.

We also compare our tracker on VOT2016 [45], VOT2018 [46], and VOT2019 [47] by adopting Expected Average Overlap (EAO), Accuracy, and Robustness as metrics, and by using the official VOT toolkit. Unfortunately, ADNet was trained on VOT data, and we could not test our modified ADNet model on this benchmarks. Results are reported in Table 2, Table 3 and Table 4, respectively.

Among all the approaches using iterative bounding box refinements, only RDNet reports results on VOT2016. As shown in Table 2, our method shows improvements over RDNet in all the three metrics.

Overall, the comparison among algorithms adopting iterative bounding box refinements confirms that dealing with conflicting transformations and allowing multiple refinements at each iteration helps improve the tracking results. We also note that the performance of some algorithms such as SiamRPN++, ATOM has decreased from VOT-2016 to VOT-2018. For instance, accuracy of SiamRPN++ is 0.64 and 0.59 on VOT-2016 and VOT-2018, respectively. As for ATOM, accuracy is 0.61 and 0.6 on VOT-2016 and VOT-2018, respectively. The accuracy of the proposed algorithm is 0.557 and 0,56 on VOT-2016 and VOT-2018, respectively. Therefore, despite accuracy slightly decreased also for our method, these results suggest that our method improves in terms of stability.

## 6. Conclusions and Future Work

This work focused on tracking strategies where the target bounding box is refined iteratively by applying a sequence of transformations. It proposed a novel formulation such that, given an image patch based on the currently estimated target bounding box, the model returns a set of *N* probability distributions over bounding box transformations. The method can apply multiple non-conflicting refinements at each iteration without introducing ambiguity during learning, i.e., without giving priority to some transformations over the others.

Experimental results show that the proposed iterative multi-refinement approach is superior to the single-refinement one, independently of the model/training strategies adopted. Overall, the proposed approach is competitive with respect to other state-of-the-art approaches that iteratively refine the target bounding box. In future work, we will try to improve the offline training procedure, as it largely impacts the tracking results. In particular, we plan to adopt a meta-learning approach to accelerate the learning of the model parameters. 

## Figures and Tables

**Table 1:** Comparison on OTB-100. Iter indicates approaches using iterative refinements. *P(20px)* indicates the mean precisions when the location error threshold is 20 pixels. AUC of IoU is the area under the curve of the success plot computed by considering the IoU values.

Algorithm	P(20px)	AUC of IoU	FPS	Iter
Retina-MAML [26]	0.926	0.712	40	
VITAL [24]	0.918	0.682	2	
SiamRPN++ [41]	0.915	0.696	35	
ECO [42]	0.910	0.691	8	
MDNet [4]	0.909	0.678	1	
RDNet [7]	0.903	0.673	4	x
**ADNet_MT (ours)**	0.895	0.660	5	x
Hier. T. [40]	0.894	0.651	23	x
IADNET [35]	0.894	0.651	3	x
ADNet [6]	0.88	0.646	2	x
ATOM [28]	0.879	0.667	30	
**Ours_MT_MD**	0.879	0.626	5	x
TSAS [36]	0.861	0.651	20	x
ACT [11]	0.855	0.622	30	
TD3T [43]	0.821	0.616	23	
A3CTD [44]	0.717	0.535	50	
GOTURN [22]	0.565	0.425	125	

**Table 2:** Comparison on VOT-2016.

Algorithm	EAO	Accuracy	Robustness
D3S [31]	0.493	0.66	0.131
UpdateNet [48]	0.481	0.61	0.21
SiamRPN++ [41]	0.464	0.64	0.20
DiMP-50 [49]	0.440	0.597	0.153
SPM [50]	0.434	0.62	0.21
ATOM [28]	0.43	0.61	0.18
ECO [42]	0.374	0.54	0.72
**Ours_MT_MD**	0.372	0.557	0.53
RDNet [7]	0.364	0.54	0.72
CCOT [51]	0.331	0.54	0.238

**Table 3:** Comparison on VOT-2018.

Algorithm	EAO	Accuracy	Robustness
D3S [31]	0.489	0.64	0.15
Ocean-off [52]	0.467	0.598	0.169
Retina-MAML [26]	0.452	0.604	0.159
DiMP-50 [49]	0.440	0.597	0.153
SiamRPN++ [41]	0.414	0.600	0.234
ATOM [28]	0.401	0.590	0.204
UPDT [53]	0.378	0.536	0.184
**Ours_MT_MD**	0.372	0.56	0.44
DRT [54]	0.356	0.519	0.201

**Table 4:** Comparison on VOT-2019.

Algorithm	EAO	Accuracy	Robustness
Retina-MAML [26]	0.313	0.57	0.366
ATOM [28]	0.292	0.603	0.411
SiamRPN++ [41]	0.292	0.58	0.446
SiamMask [29]	0.287	0.594	0.461
**Ours_MT_MD**	0.232	0.513	0.72

## Data Availability

Not applicable.
